# Association between infant breastfeeding practices and timing of peak height velocity: A nationwide longitudinal survey in Japan

**DOI:** 10.1038/s41390-023-02706-y

**Published:** 2023-07-03

**Authors:** Yousuke Higuchi, Naomi Matsumoto, Shintaro Fujiwara, Yuki Ebuchi, Mahoko Furujo, Kazue Nakamura, Toshihide Kubo, Takashi Yorifuji

**Affiliations:** 1 Department of Pediatrics, National Hospital Organization Okayama Medical Center, 1711-1 Tamasu, Kita-ku, Okayama 701-1192 Japan; 2 Department of Epidemiology, Graduate School of Medicine, Dentistry and Pharmaceutical Sciences, Okayama University, 2-5-1 Shikata-cho, Kita-ku, Okayama 700-8558 Japan; 3 Division of Neonatology, National Hospital Organization Okayama Medical Center, 1711-1 Tamasu, Kita-ku, Okayama 701-1192 Japan

## Abstract

**Background:**

Several studies have discovered an association between infant feeding practices and puberty timing; however, most have involved female cohorts. We investigated the association between infant feeding practices and the timing of peak height velocity in boys and girls.

**Methods:**

Data on infant feeding methods and anthropometric measurements were collected from a nationwide Japanese birth cohort study. The age at peak height velocity (APV, years) was estimated and compared. Subsequently, the effects of breastfeeding duration were analyzed.

**Results:**

Of the 13,074 eligible participants, 650, 9455, and 2969 were formula-, mixed-, and exclusively breastfed, respectively. Among girls, the mean APV was significantly later in the mixed-fed (standardized regression coefficient (β): 0.094, 95% confidence interval (CI): 0.004–0.180) and exclusively breastfed (β: 0.150, 95% CI: 0.056–0.250) groups than in the formula-fed group. Among boys, the mean APV was not significantly different among the three groups; however, a sensitivity analysis that excluded preterm birth revealed more significantly delayed APV in the breastfed-only group compared to the formula-fed group. Furthermore, a multiple linear regression model revealed that a longer breastfeeding period was associated with later APV.

**Conclusions:**

Infant breastfeeding practices can affect the timing of peak height velocity in both boys and girls.

**Impact:**

- Several studies have discovered an association between infant feeding practices and puberty timing; however, most have involved female cohorts. Age at peak height velocity, derived from longitudinal height measurements, is a useful marker of secondary sexual maturity milestones in boys and girls.
- A Japanese birth cohort study revealed that breastfed children had a later age at peak height velocity than their formula-fed counterparts; this was more prominent among girls than boys.
- Furthermore, a duration-effect relationship was observed, where longer breastfeeding duration was associated with a later age at peak height velocity.

## Introduction

Puberty is the period of transition from childhood to adulthood and sexual maturity, during which bodily changes occur. The initial manifestations of secondary sexual characteristics are breast development in girls and increased testicular volume in boys. Another major change is a growth spurt, in which a marked acceleration in stature occurs during the early stages of puberty; approximately 20% of an adult’s height is gained during this period. The age at peak height velocity (APV) differs between sexes and is approximately 2 years and 1.5 years after puberty onset in boys and girls, respectively.

The timing and progression of puberty are affected by various factors, including race/ethnicity, genetics, psychosocial environment, endocrine-disrupting chemicals, and nutrition.^1^ Moreover, perinatal factors, such as maternal smoking, preterm birth, or small for gestational age (SGA) birth, may also predispose adolescents to early puberty onset.^1–5^ Several studies have discovered an association between infant feeding practices and puberty timing; however, most have involved female cohorts.^6–8^ A recent study revealed that girls who were not breastfed experienced an earlier onset of breast and pubic hair development than those who were breastfed for ≥6 months after adjusting for prepubertal body mass index (BMI).^9^ However, this association was insignificant among Asian/Pacific Islanders, possibly due to a smaller sample size, and it was not examined in boys.

The age of pubertal onset has decreased globally and has become a concern.^10^ A previous meta-analysis worldwide revealed that the age of breast development onset has decreased by 0.24 years per decade over the past 36 years.^11^ This trend toward earlier pubertal milestones can be partly attributed to the increasing prevalence of childhood obesity.^12,13^ In contrast, the timing of puberty onset and its association with obesity in boys remains controversial.^12,14–18^ This may be because secondary sexual characteristics in girls (such as breast enlargement or menarche) are easily recognized. In contrast, the onset of testicular enlargement is less conspicuous, and the cracking of the voice involves a relatively subjective evaluation, rendering the accurate estimation of the timing of puberty among boys more challenging.

We aimed to investigate the effect of infant breastfeeding practices on the timing of peak height velocity in boys and girls using data from a Nationwide Longitudinal Survey in Japan. We hypothesized that breastfed children would have a later peak height velocity onset at puberty than formula- or mixed-fed children.

## Methods

### Ethical considerations

All procedures in this study were performed in accordance with the 1964 Declaration of Helsinki and the 2003 Japanese Ethical Guidelines for Clinical Research and its subsequent amendments. This study was approved by the Institutional Review Boards of Okayama University Hospital (1506–073) and Okayama Medical Center (2019–027).

### Participants

The Japanese Ministry of Health, Labour, and Welfare has conducted the “Longitudinal Survey of Newborns in the 21st Century” since 2001. This birth cohort investigation annually collected information on family circumstances, child-rearing, and children’s health and developmental statuses from all families in Japan to whom infants were born between January 10 and 17 and between July 10 and 17, 2001. The first set of questionnaires were mailed to 53,575 families when eligible infants reached the age of 6 months and were completed by 47,015 families (response rate, 88%). The form included questions regarding children’s perinatal status and household and socioeconomic factors such as residential area, maternal academic achievement level, and smoking status. Almost all (>98%) of the parents were Japanese, whereas most of the remainder were of Asian descent, reflecting the Japanese demographic composition at that time. Follow-up questionnaires including each child’s height and weight were sent at ages 1.5, 2.5, 3.5, 4.5, 5.5, 7, 8, 9, 10, 11, 12, 13, 14, and 15 years. Birth records from Japanese vital statistics, including birth weight, gestational age, single or multiple births, sex, parity, and maternal age at delivery, were retrieved and linked to the data. SGA, appropriate for gestational age (AGA), and large for gestational age (LGA) were defined as sex- and parity-specific birth weights below the 10th percentile, between the 10th and 90th percentiles, and above the 90th percentile for gestational age, respectively, based on the Japanese population reference. Maternal smoking status and educational attainment were obtained from the first and second surveys, respectively.

### Infant feeding practices and anthropometric measurements

We obtained infant feeding methods and breastfeeding duration data from surveys at 6 months; the feeding methods were classified into three types: formula-fed (never breastfed other than colostrum, if applicable), mixed-fed (breastfeeding combined with formula), or exclusively breastfed. Children in the “exclusively breastfed” group were never formula-fed during the first 6–7 months of life. Heights and weights from the ages of 5.5 to 15 years were collected (the months these measurements were obtained). In Japan, children’s heights and weights are usually measured every semester for 15 years during the preschool and school years.

### Data cleaning and analysis

Participants for whom data on all 10 height measurements (acquired over as many years), weight at 5.5 years, and infant feeding practice were available were included in the analysis. The data were cleaned to reduce the possibility of erroneous height values: height at age 5.5 years was defined as HT1, height at age 7 years as HT2, and so on until height at 15 years (HT10). Participants who did not satisfy the following formulae were excluded:

Boys: HT1 < HT2 < HT3 < HT4 < HT5 < HT6 < HT7 < HT8, HT8 < HT9 + 5, and HT9 < HT10 + 5

Girls: HT1 < HT2 < HT3 < HT4 < HT5 < HT6, HT6 < HT7 + 5, HT7 < HT8 + 5, HT8 < HT9 + 5, and HT9 < HT10 + 5

These formulae were set up to allow for monotonic increments in height, avoid duplication until late puberty stage, and remain within the maximum allowed measurement error or diurnal variation after reaching near-adult height.^19^ The thresholds for monotonic increment limits were set based on the Japanese population; −2 standard deviations ≤ APV ≤ first quartile had a near-adult height at approximately 14 years for boys and 12 years for girls.^20^

We used the SuperImposition by Translation and Rotation (SITAR) growth curve model to calculate the APV.^21^ SITAR is a non-linear multilevel model with natural cubic splines that estimates the population average growth curve and random effects; it captures differences in size, tempo, and velocity, thereby explaining an individual’s growth trajectory. The SITAR model allows flexibility in fitting spline curves by adjusting the degrees of freedom (for which we adopted 5 for both sexes).^5^ The variance score explained by the SITAR model was calculated. We obtained each participant’s estimated APV from the SITAR model with the “sitar” package (version 1.2.0) and the “iapvbs” package (version 0.0.2).^22^ The later package outputs the estimated APV with a flag value: 0 represents a normal estimated APV, 1 represents an estimated APV that is significantly close to the minimum or maximum age measurement, 2 represents an estimated APV equal to the minimum or maximum age measurement, 3 represents an estimated APV outside the range of age measurements, and 4 represents an estimated APV that is questionable owing to some other reasons.

### Statistical analyses

Maternal and child characteristics and socioeconomic status were confounding factors based on the existing literature.^3–8^ Child factors included sex (dichotomous), birth weight (<2500, 2500–4000, and >4000 g, categorical), weight for gestational age (SGA, AGA, and LGA, categorical), term or preterm birth (<37 weeks’ gestation, dichotomous), and singleton or multiple births (dichotomous). Maternal factors included maternal age at delivery (<30, 30–34, and ≥35 years, categorical), smoking habits (nonsmoker, <10 daily cigarettes, and ≥10 daily cigarettes, categorical), maternal educational level (categorical), and residential area where the participant was born (ward, city, and town or village, categorical). We reclassified the original eight education categories into four: junior high school and others, high school, junior college (2 years) or vocational school, and university (4 years) or higher. In a previous study of the same population, almost all participants of both sexes who were overweight or obese at the age of 15 years had experienced adiposity rebound before 4.5 years; as such, we adopted the BMI at 5.5 years as the prepubertal BMI.^23^

Multiple linear regression was used to estimate the relationships after adjusting for the above confounders with and without prepubertal BMI, and the formula-fed group was used as a reference. To evaluate the association with preterm birth, we excluded participants with preterm births (*n* = 604) from the sensitivity analysis. In the additional linear regression analyses of the breastfeeding duration-effect relationship, we subcategorized infant feeding types into exclusively formula-fed, formula-fed plus only colostrum, breastfed (combined with formula) for 1–2 months, 3–5 months, and 6–7 months, and exclusively breastfed at 6–7 months. The main analysis was performed using the R statistical software (R Foundation, Vienna, Austria, version 4.1.2) through R Studio (RStudio, Boston, America, 2021.09.1 + 372 “Ghost Orchid”). The statistical analyses were performed using Stata 17 (StataCorp). A *p* < 0.05 was considered statistically significant.

## Results

**Fig. 1:**
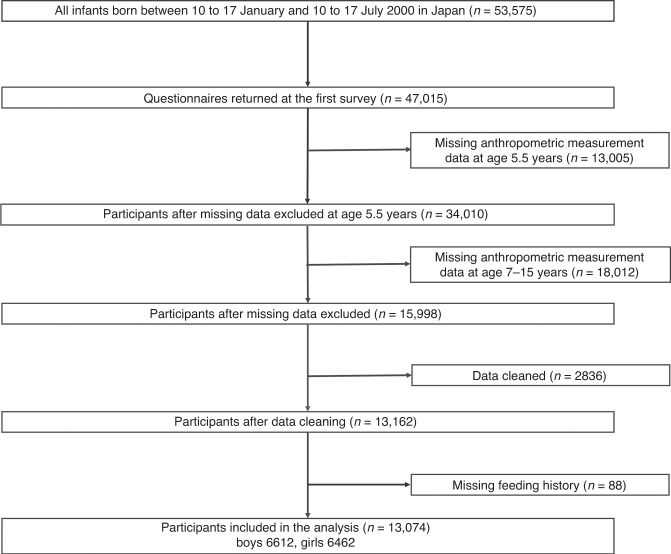
Flowchart of the study participant selection process.

Among the Longitudinal Survey of Newborns in the 21st Century cohort, 15,998 (29.9%) participants had complete anthropometric measurement data available for the analysis period. The data-cleaning process reduced the number of participants to 13,162; the variance scores attained by the aforementioned model increased from 89.59 to 92.93 for boys and 89.55 to 93.08 for girls. After excluding participants lacking data on feeding practice history, we finally included 13,074 participants (Fig. 1), 98.7% of whom were Japanese parents. The baseline characteristics of eligible and ineligible participants were similar, except for fewer maternal smoking habits and higher maternal educational attainment among the eligible population (Table 1).

**Table 1:** Demographic characteristics of entire participants categorized by study eligibility.

Background characteristic			
	Excluded population	Analysis population	Entire cohort
Feeding practice, *n* (%)			
Formula-fed	2282 (6.8)	650 (5.0)	2932 (6.3)
Mixed-fed	24428 (72.8)	9455 (72.3)	33883 (72.7)
Exclusively breastfed	6832 (20.4)	2969 (22.7)	9801 (21.0)
Child characteristics			
Sex, *n* (%)			
Male	17780 (52.5)	6645 (50.6)	24425 (52.0)
Female	16095 (47.5)	6495 (49.4)	22590 (48.0)
Birth weight, *n* (%)			
2500–4000 g	30526 (90.1)	11935 (90.8)	42461 (90.3)
<2500 g	2929 (8.7)	1076 (8.2)	4005 (8.5)
≥4000 g	406 (1.2)	129 (1.0)	535 (1.1)
N/A	14 (0.0)	0 (0.0)	14 (0.0)
Weight for gestational age, *n* (%)			
Appropriate for gestational age	26968 (80.5)	10504 (80.8)	37472 (80.6)
Small for gestational age	2721 (8.1)	1017 (7.8)	3738 (8.0)
Large for gestational age	3823 (11.4)	1472 (11.3)	5295 (11.4)
Gestation time, *n* (%)			
Term birth	32100 (94.8)	12533 (95.4)	44633 (94.9)
Preterm birth	1775 (5.2)	607 (4.6)	2382 (5.1)
Delivery, *n* (%)			
Singleton birth	33162 (97.9)	12877 (98.0)	46039 (97.9)
Multiple birth	713 (2.1)	263 (2.0)	976 (2.1)
Maternal characteristics			
Age at delivery, *n* (%)			
<25 years	5277 (15.6)	891 (6.8)	6168 (13.1)
25–35 years	24384 (71.2)	10195 (77.6)	34579 (73.6)
≥35 years	4214 (12.4)	2054 (15.6)	6268 (13.3)
Smoking habit, *n* (%)			
None	26759 (79.0)	11806 (89.9)	38565 (82.0)
<10/day	1886 (5.6)	418 (3.2)	2304 (4.9)
≥10/day	4949 (14.6)	871 (6.6)	5820 (12.4)
N/A	281 (0.8)	45 (0.3)	326 (0.7)
Educational level, *n* (%)			
University or higher	3711 (11.0)	2320 (17.7)	6031 (12.8)
Junior college or vocational school	12113 (35.8)	5921 (45.1)	18034 (38.4)
High school	12707 (37.5)	4450 (33.9)	17157 (36.5)
Junior high school or others	2124 (6.3)	329 (2.5)	2453 (5.2)
N/A	3220 (9.5)	120 (0.9)	3340 (7.1)
Residential area, *n* (%)			
Ward	7182 (21.2)	2879 (21.9)	10061 (21.4)
City	20047 (59.2)	7896 (60.1)	27943 (59.4)
Town or village	6646 (19.6)	2365 (18.0)	9011 (19.2)

*N/A* not applicable.

Among the 13,074 participants, 650 (5.0%) were formula-fed, 9455 (72.3%) were mixed-fed, and 2969 (22.7%) were exclusively breastfed at 6–7 months of age. Twenty-six boys and one girl were identified as Flag 1, 103 boys as Flag 2, and none as Flag 3 or 4. The demographic characteristics of the participants and their APVs, sorted according to infant feeding practices, are presented in Table 2. The APVs distribution chart in boys and girls are shown in Supplementary Fig. 1.

**Table 2:** Demographic characteristics of study participants categorized by infant feeding practices.

Background characteristic	Formula-fed	Mixed-fed	Exclusively breastfed
	(*n* = 650)	(*n* = 9455)	(*n* = 2969)
Child characteristics			
Sex, *n* (%)			
Male	331 (50.9)	4808 (50.9)	1473 (49.6)
Female	319 (49.1)	4647 (49.2)	1496 (50.4)
Birth weight, *n* (%)			
2500–4000 g	563 (86.6)	8510 (90.0)	2801 (94.3)
<2500 g	83 (12.8)	851 (9.0)	139 (4.7)
≥4000 g	4 (0.6)	139 (4.7)	127 (1.0)
Weight for gestational age, *n* (%)			
Appropriate for gestational age	505 (79.0)	7510 (80.3)	2436 (82.9)
Small for gestational age	63 (9.9)	773 (8.3)	174 (5.9)
Large for gestational age	71 (11.1)	1065 (11.4)	330 (11.2)
Gestation time, *n* (%)			
Term birth	600 (92.3)	8968 (94.9)	2902 (97.7)
Preterm birth	50 (7.7)	487 (5.2)	67 (2.3)
Delivery, *n* (%)			
Singleton birth	33 (5.1)	229 (2.4)	263 (2.0)
Multiple birth	617 (94.9)	9226 (97.6)	12811 (98.0)
Prepubertal BMI, mean (SD)	15.46 (1.55)	15.38 (1.42)	15.41 (1.37)
Maternal characteristics			
Age at delivery, *n* (%)			
<25 years	50 (7.7)	672 (7.1)	163 (5.5)
25–35 years	455 (70.0)	7313 (77.4)	2377 (80.1)
≥35 years	145 (22.3)	1470 (15.6)	429 (14.5)
Smoking habit, *n* (%)			
None	542 (83.4)	8395 (88.8)	2812 (94.7)
<10/day	23 (3.5)	325 (3.4)	68 (2.3)
≥10/day	81 (12.5)	700 (7.4)	84 (2.8)
N/A	4 (0.6)	35 (0.4)	5 (0.2)
Educational level, *n* (%)			
University or higher	52 (8.0)	1630 (17.2)	629 (21.2)
Junior college or vocational school	233 (35.9)	4246 (44.9)	1419 (47.8)
High school	314 (48.3)	3242 (34.3)	863 (29.1)
Junior high school or others	41 (6.3)	252 (2.7)	35 (1.2)
N/A	10 (1.5)	85 (0.9)	23 (0.8)
Residential area, *n* (%)			
Ward	118 (18.2)	2079 (22.0)	669 (21.9)
City	397 (61.1)	5694 (60.2)	1764 (59.4)
Town or village	135 (20.8)	1682 (17.8)	536 (18.1)
Age at peak height velocity, male mean (SD)	12.63 (0.91)	12.72 (0.90)	12.76 (0.90)
Age at peak height velocity, female mean (SD)	10.70 (0.79)	10.82 (0.80)	10.89 (0.82)

*BMI* body mass index, *N/A* not applicable, *SD* standard deviation.

The results of multiple linear regression analyses of infant feeding practices and APV, adjusted for child- and maternal-related factors, are presented in Table 3. After adjusting for covariates, mixed feeding and breastfeeding were associated with later APV among the girls. Additionally, a higher prepubertal BMI was negatively correlated with the APV in boys (standardized regression coefficient (β): −0.099, *p* < 0.001, 95% confidence interval (CI): −0.11 to −0.083) and girls (β: −0.15, *p* < 0.001, 95% CI: −0.16 to −0.13). Excluding prepubertal BMI analysis was also consistent with the result (mixed-fed [β: 0.11, 95% CI: 0.039–0.180] and exclusively breastfed groups [β: 0.15, 95% CI: 0.081–0.230] had higher values compared with the formula-fed group [both sexes combined]). Our analysis also revealed that SGA birth, smoking ≥10 cigarettes per day, and maternal age ≥25 years at delivery were positively associated with early APV among boys; however, only SGA birth was significantly associated with early APV among girls. Sensitivity analysis, which excluded preterm infants, revealed that mixed-fed and breastfed infants had a later APV than formula-fed infants, including boys (Table 4).

**Table 3:** Association between infant feeding practices and the APV among participants stratified by sex.

	Boys (*n* = 6539)					Girls (*n* = 6388)				
APV	β*	Standard error	95% CI lower	95% CI upper	*P*- value	β*	Standard error	95% CI lower	95% CI upper	*P*- value
Formula-fed	(Reference)					(Reference)				
Mixed-fed	0.069	0.051	–0.031	0.170	0.180	0.094	0.046	0.004	0.180	0.041
Exclusively breastfed	0.092	0.055	–0.016	0.200	0.100	0.150	0.049	0.056	0.250	0.002
Child characteristics										
Birth weight										
2500–4000 g	(Reference)					(Reference)				
<2500 g	-0.140	0.050	–0.240	–0.042	0.005	–0.071	0.041	–0.150	0.009	0.084
≥4000 g	0.023	0.110	–0.180	0.230	0.830	0.140	0.120	–0.100	0.380	0.260
Weight for gestational age										
Appropriate for gestational age	(Reference)					(Reference)				
Small for gestational age	–0.094	0.046	–0.180	–0.004	0.040	–0.140	0.043	–0.220	–0.050	0.002
Large for gestational age	0.098	0.036	0.027	0.170	0.007	0.048	0.032	–0.015	0.110	0.140
Delivery										
Singleton birth	(Reference)					(Reference)				
Multiple birth	0.150	0.082	–0.007	0.310	0.061	0.220	0.073	0.081	0.370	0.002
Prepubertal BMI	–0.099	0.008	–0.110	–0.083	<0.001	–0.150	0.007	–0.160	–0.130	<0.001
Maternal characteristics										
Age at delivery										
<25 years	(Reference)					(Reference)				
25–35 years	–0.120	0.044	–0.200	–0.030	0.008	0.019	0.040	–0.059	0.096	0.640
≥35 years	–0.150	0.051	–0.250	–0.046	0.004	–0.029	0.045	–0.120	0.060	0.520
Smoking habit										
None	(Reference)					(Reference)				
<10 cigarettes/day	–0.026	0.064	–0.150	0.100	0.690	–0.059	0.055	–0.170	0.048	0.280
≥10 cigarettes/day	–0.150	0.046	–0.240	–0.063	0.001	–0.055	0.040	–0.130	0.023	0.170
N/A	–0.630	0.190	–1.000	–0.026	0.001	–0.043	0.170	–0.380	0.290	0.800
Educational level										
University or higher	(Reference)					(Reference)				
Junior college or vocational school	–0.033	0.031	–0.094	0.028	0.290	0.018	0.027	–0.035	0.071	0.510
High school	–0.033	0.033	–0.097	0.032	0.320	-0.017	0.029	–0.074	0.040	0.570
Junior high school or others	–0.150	0.078	–0.300	0.007	0.062	–0.120	0.066	–0.250	0.013	0.080
N/A	0.025	0.110	–0.190	0.240	0.820	0.024	0.120	–0.210	0.260	0.850
Residential area										
Ward	(Reference)					(Reference)				
City	0.005	0.028	–0.049	0.060	0.850	–0.083	0.024	–0.130	–0.036	0.001
Town or village	0.013	0.035	–0.056	0.081	0.710	–0.120	0.031	–0.180	–0.055	<0.001

*APV* age at peak height velocity, *BMI* body mass index, *CI* confidence interval, *N/A* not applicable.

*Standardized regression coefficient.

**Table 4:** Sensitivity analysis of the association between infant feeding practices and the APV (excluding 604 preterm births) stratified by sex.

	Boys (*n* = 6189)					Girls (*n* = 6135)				
APV	β*	Standard error	95% CI lower	95% CI upper	*P*-value	β*	Standard error	95% CI lower	95% CI upper	*P*-value
Formula-fed	(Reference)					(Reference)				
Mixed-fed	0.010	0.053	0.000	0.210	0.051	0.110	0.048	0.011	0.200	0.028
Exclusively breastfed	0.130	0.056	0.020	0.240	0.020	0.160	0.051	0.064	0.260	0.001
Child characteristics										
Birth weight										
2500–4000 g	(Reference)					(Reference)				
<2500 g	–0.110	0.067	–0.250	0.017	0.089	–0.080	0.049	–0.180	0.015	0.100
≥4000 g	–0.027	0.110	–0.230	0.180	0.800	0.140	0.033	–0.100	0.390	0.250
Weight for gestational age										
Appropriate for gestational age	(Reference)					(Reference)				
Small for gestational age	–0.094	0.050	–0.190	0.005	0.063	–0.120	0.047	–0.210	–0.027	0.011
Large for gestational age	0.120	0.037	0.052	0.200	0.001	0.044	0.033	–0.021	0.110	0.180
Delivery										
Singleton birth	(Reference)					(Reference)				
Multiple birth	0.096	0.110	–0.120	0.310	0.390	0.260	0.094	0.080	0.450	0.005
Prepubertal BMI	–0.100	0.008	–0.120	–0.086	<0.001	–0.150	0.007	–0.160	–0.130	<0.001
Maternal characteristics										
Age at delivery										
<25 years	(Reference)					(Reference)				
25–35 years	–0.110	0.045	–0.200	–0.025	0.012	0.012	0.041	–0.068	0.091	0.770
≥35 years	–0.130	0.052	–0.240	–0.032	0.010	–0.031	0.047	–0.120	0.061	0.510
Smoking habit										
None	(Reference)					(Reference)				
<10 cigarettes/day	–0.020	0.065	–0.150	0.110	0.760	–0.069	0.056	–0.170	0.040	0.210
≥10 cigarettes/day	–0.150	0.047	–0.240	–0.059	0.001	–0.059	0.041	–0.140	0.021	0.150
N/A	–0.590	0.200	–0.990	–0.200	0.003	–0.035	0.170	–0.380	0.310	0.840
Educational level										
University or higher	(Reference)					(Reference)				
Junior college or vocational school	–0.050	0.032	–0.110	0.013	0.120	0.023	0.028	–0.032	0.077	0.420
High school	–0.051	0.034	–0.120	0.015	0.130	-0.011	0.030	–0.069	0.047	0.710
Junior high school or others	–0.110	0.080	–0.270	0.047	0.170	–0.120	0.068	–0.260	0.011	0.073
N/A	–0.047	0.110	–0.270	0.170	0.670	0.043	0.120	–0.200	0.290	0.730
Residential area										
Ward	(Reference)					(Reference)				
City	0.000	0.028	–0.055	0.056	1.000	–0.086	0.024	–0.130	–0.037	0.001
Town or village	0.010	0.036	–0.06	0.080	0.760	–0.110	0.032	–0.170	–0.043	0.001

*APV* age at peak height velocity, *BMI* body mass index, *CI* confidence interval, *N/A* not applicable.

*Standardized regression coefficient.

Furthermore, linear regression analysis using breastfeeding duration as the exposure revealed a duration-effect relationship, with a later APV associated with a longer breastfeeding duration (Table 5). In addition, sub-analysis (formula-fed with/without colostrum group as a reference) revealed similar results (Supplementary Table 1). The variance inflation factors for Tables 3, 5, and Supplementary Table 1 are shown in Supplementary Fig. 2.

**Table 5:** Association between duration of breastfeeding and the APV.

APV	β*	Standard error	95% CI lower	95% CI upper	*P*-value
Formula-fed (*n* = 171)	(Reference)				
Formula-fed with only colostrum (*n* = 479)	0.060	0.075	–0.087	0.210	0.420
Breastfed 1–2 months (*n* = 2413)	0.082	0.067	–0.049	0.210	0.220
3–5 months (*n* = 2455)	0.120	0.067	–0.010	0.250	0.070
6–7 months (*n* = 4770)	0.150	0.066	0.018	0.280	0.026
Exclusively breastfed (*n* = 2969)	0.170	0.066	0.038	0.300	0.012
Child characteristics					
Sex					
Male	(Reference)				
Female	–1.900	0.015	–1.930	–1.870	<0.001
Birth weight					
2500–4000 g	(Reference)				
<2500 g	–0.096	0.032	–0.160	–0.033	0.003
≥4000 g	0.086	0.080	–0.070	0.240	0.280
Weight					
Appropriate for gestational age	(Reference)				
Small for gestational age	–0.120	0.032	–0.180	–0.050	<0.001
Large for gestational age	0.071	0.024	0.024	0.120	0.003
Delivery					
Singleton birth	(Reference)				
Multiple birth	0.190	0.056	0.081	0.300	0.001
Prepubertal BMI	–0.120	0.005	–0.130	–0.110	<0.001
Maternal characteristics					
Age at delivery					
<25 years	(Reference)				
25–35 years	–0.050	0.030	–0.110	0.009	0.095
≥35 years	–0.088	0.034	–0.160	–0.021	0.010
Smoking habit					
None	(Reference)				
<10/day	–0.039	0.042	–0.120	0.043	0.350
≥10/day	–0.087	0.031	–0.150	–0.027	0.005
N/A	–0.330	0.130	–0.580	–0.080	0.010
Educational level					
University or higher	(Reference)				
Junior college or vocational school	–0.003	0.021	–0.044	0.037	0.870
High school	–0.019	0.022	–0.030	0.024	0.380
Junior high school or others	–0.120	0.050	–0.220	–0.022	0.016
N/A	0.058	0.080	–0.100	0.220	0.470
Residential area					
Ward	(Reference)				
City	–0.036	0.018	–0.072	0.000	0.050
Town or village	–0.047	0.024	–0.093	0.000	0.047

*APV* age at peak height velocity, *BMI* body mass index, *CI* confidence interval, *N/A* not applicable.

*Standardized regression coefficient.

## Discussion

In this nationwide birth cohort study, we observed that infant breastfeeding practices affected the timing of peak height velocity. Breastfed children had later APVs than their formula-fed counterparts; this was more pronounced among girls than boys. In the sensitivity analysis, excluding preterm birth data strengthened these associations; they became significant among boys. Furthermore, multiple regression analysis revealed that longer breastfeeding duration was associated with later APV.

It is important to consider racial disparity in pubertal development. A prospective cohort study from the United States discovered that thelarche onset for girls who were exclusively formula-fed was approximately 2.5 months earlier than in those who were breastfed for more than 6 months. However, the association was only evident among African Americans.^9^ Another study in the United States that revealed similar results included a small number of Asians (57 out of 1237 study participants; 15 were predominantly breastfed, 33 were mixed-fed, and six were formula-fed).^8^ An observational study from Korea discovered that early puberty occurred less frequently in children who were breastfed for 6 months or longer than in those who were not (odds ratio: 0.37; 95% CI: 0.18–0.74); however, the participants were not stratified by sex.^24^ A study of Chinese children conducted in Hong Kong discovered that breastfeeding was not associated with pubertal timing. However, this study defined breastfed infants as those breastfed for at least 3 months.^25^ Our cohort, composed almost entirely of Japanese individuals, revealed a significant difference in APV among those breastfed for 6 months or longer. As exclusive breastfeeding is recommended by the World Health Organization and United Nations Children’s Fund for the first 6 months of life, defining breastfeeding using the interval endorsed by these agencies should be more appropriate.^26,27^ Furthermore, a longer and more frequent mother-infant skin-to-skin contact may affect the APV; however, no information was collected on breastfeeding style (direct breastfeeding or bottle-feeding pumped breastmilk).^28,29^ Some studies that support the beneficial outcomes of breastfeeding may be subject to publication bias; ^30^however, our findings were consistent with those of other investigators.

In our previous analysis of the same population, exclusive breastfeeding at 6–7 months was associated with a lower risk of overweight/obesity at 7 years than formula feeding.^31^ Another recent study revealed that early adiposity rebound was associated with overweight/obesity at 15 years and that the APV was earlier in the overweight/obese groups than in the normal-weight group.^23^ Our current study expanded on the associations between infant feeding practices and pubertal timing, particularly after considering potential confounding factors, such as prepubertal BMI and perinatal circumstances. A recent prospective birth cohort study of predominantly Black mothers and children using the SITAR model also revealed that obesity at 5–7 years was associated with an earlier APV in boys, whereas overweight/obesity at 5–7 years was associated with an earlier APV in girls.^32^ The association between infant feeding practices and puberty has been confounded by obesity, especially in girls. This study revealed an exposure-response relationship between breastfeeding duration and APV, regardless of sex, after adjusting for prepubertal BMI, suggesting a direct effect of infant feeding practices on puberty.

A recent large cohort study investigated the association between breastfeeding and pubertal signs stratified by sex.^33^ The shorter duration of exclusive breastfeeding was significantly associated with earlier pubertal development in boys but not girls. Our findings were the opposite in boys and girls. Some covariates also revealed significant differences between boys and girls; however, the reason for this is unknown. SGA children tend to experience early puberty than AGA counterparts owing to catch-up growth with rapid weight gain in early childhood; this is believed to cause increased visceral adiposity, decreased insulin sensitivity, and elevated insulin-like growth factor-I levels.^34^ Our data also revealed that SGA birth was associated with earlier APVs than AGA birth. One study published in 1995 suggested an association between preterm birth and precocious puberty; however, no such association was observed in a later systematic review.^3^ A recent study of a Finnish birth cohort that used the SITAR model also revealed no association.^5^ In our sensitivity analysis, wherein preterm infants were excluded, the association between formula feeding and early puberty was reaffirmed or even stronger. A Danish national birth cohort study revealed an association between maternal prenatal smoking and early puberty.^4 ^Our analysis used information on environmental smoking exposure in infants and revealed similar results only among boys. The overall mechanism for puberty onset has not been elucidated; however, various exposures from the prenatal period to infancy can affect puberty timing, which manifests even more than 10 years later.

APV, derived from longitudinal measurements of height, is a useful marker of secondary sexual maturity milestones, such as Tanner staging or voice cracking. Frequent and precise assessment of secondary sexual characteristics is challenging, especially in a large population of children. Some studies relied on self-assessment for Tanner staging; however, objectively assessing the progression of secondary sexual development other than menarche may have been overlooked.^28,35,36^ A birth cohort study in the United Kingdom examined breastfeeding duration and puberty timing after adjusting for depression during pregnancy.^37^ Mothers with higher anxiety levels were less likely to breastfeed, and longer breastfeeding duration was associated with later menarche; however, no association was discovered between breastfeeding and voice change in males. APV is an objective measure less susceptible to subjective and recall biases.

This study had some limitations. Its longitudinal nature makes it susceptible to missing unmeasured confounders for breastfeeding and health outcomes. Parental information, such as maternal pre-pregnancy BMI and gestational diabetes mellitus, and genetic factors, such as age at maternal menarche and paternal APV, were unavailable. Prepubertal BMI can reflect genetic factors from parents and the rearing environment as confounders and an intermediate factor of exposure to breastfeeding. This significance was attenuated when the prepubertal BMI was included as a covariate. Additionally, we adjusted for maternal characteristics (smoking status and educational level); however, we could not exclude the possibility of other residual confounding factors. Moreover, we did not obtain data on the children’s adiposity or body composition measurements, which are also associated with pubertal timing.^38^ We could not rule out the possibility that children in the exclusively breastfed group could have received liquid (water and sugar beverages) or complementary food in the first survey; thus, the exclusively breastfed group might have included some children not complying with the World Health Organization and United Nations Children’s Fund recommendation strictly. Almost all study participants were Japanese, and generalizability to other populations may be limited. The reliability of anthropometric data and the validity of the data cleaning process also remain concerns. Height and weight measurements were reported by parents, which may be less reliable than obtaining these measurements from official healthcare records. Moreover, the number of children included in the analysis was constrained by the large amount of missing anthropometric data. The SITAR model can be used with a few measurements; however, we excluded participants with missing values to obtain a more accurate estimate. Additionally, data cleaning to exclude participants up to a certain age whose height decreased relative to the preceding year increased the explanatory score of the model; however, more “rigorous” cleaning, which may have increased its value even further, was not performed. No universally agreed standard methods for cleaning height measurement data currently exist.^39^

In conclusion, our data demonstrate that infant breastfeeding practices affect the timing of peak height velocity in both boys and girls. Moreover, we observed a duration-effect relationship, wherein a later APV was observed in participants who experienced a longer breastfeeding period after adjusting for perinatal and socioeconomic confounding factors.

### Supplementary information

Supplementary Figure 1

Supplementary Figure 2

Supplementary Table

## Data Availability

Restrictions apply to the availability of some or all data generated or analyzed during this study to preserve patient confidentiality or because they were used under licenses. The corresponding author will accept requests to access the data and provide details regarding the restrictions and conditions under which such access may be provided.
