# Supplementary figures and images for: Association between infant breastfeeding practices and timing of peak height velocity: A nationwide longitudinal survey in Japan
Source: Pediatr Res. 2023 Jul 3;94(5):1845–54. doi: 10.1038/s41390-023-02706-y (PMC10624627; doi:10.1038/s41390-023-02706-y)

Supplemental figure 1. The APV Distribution Chart

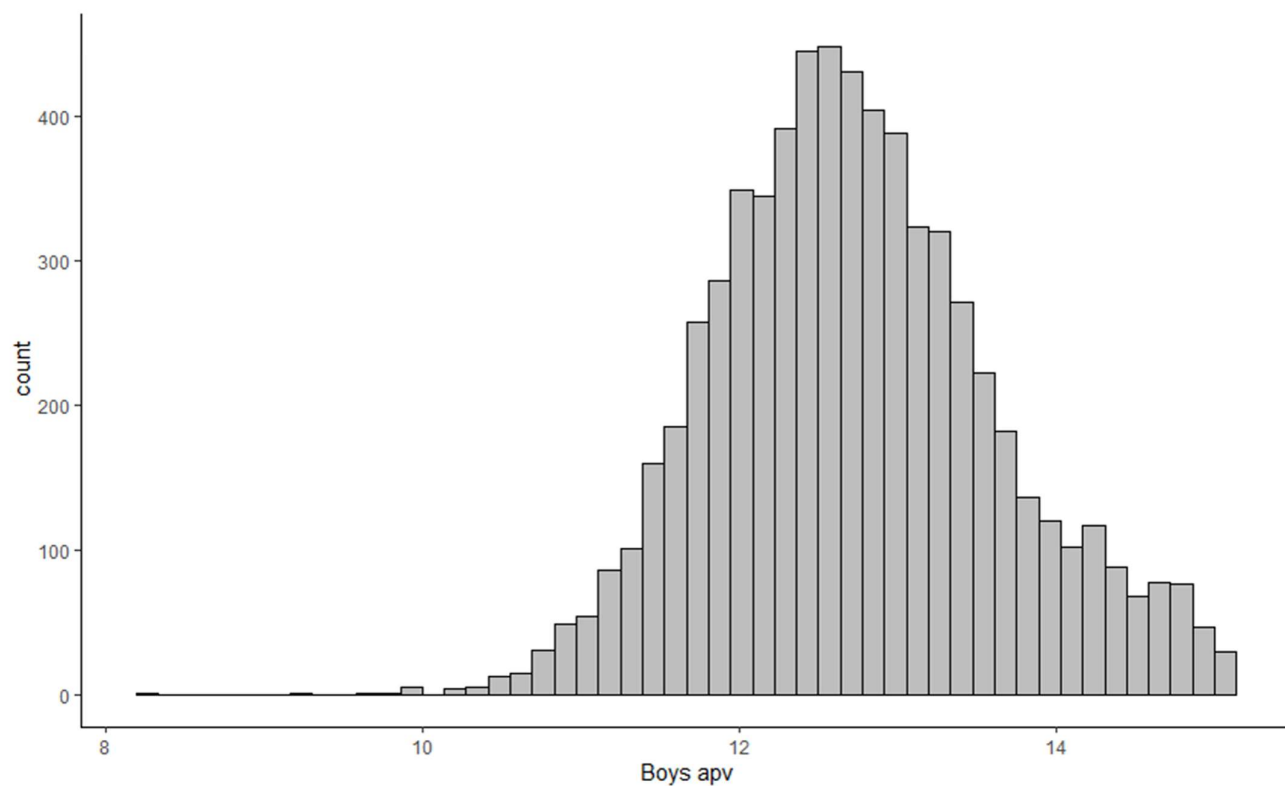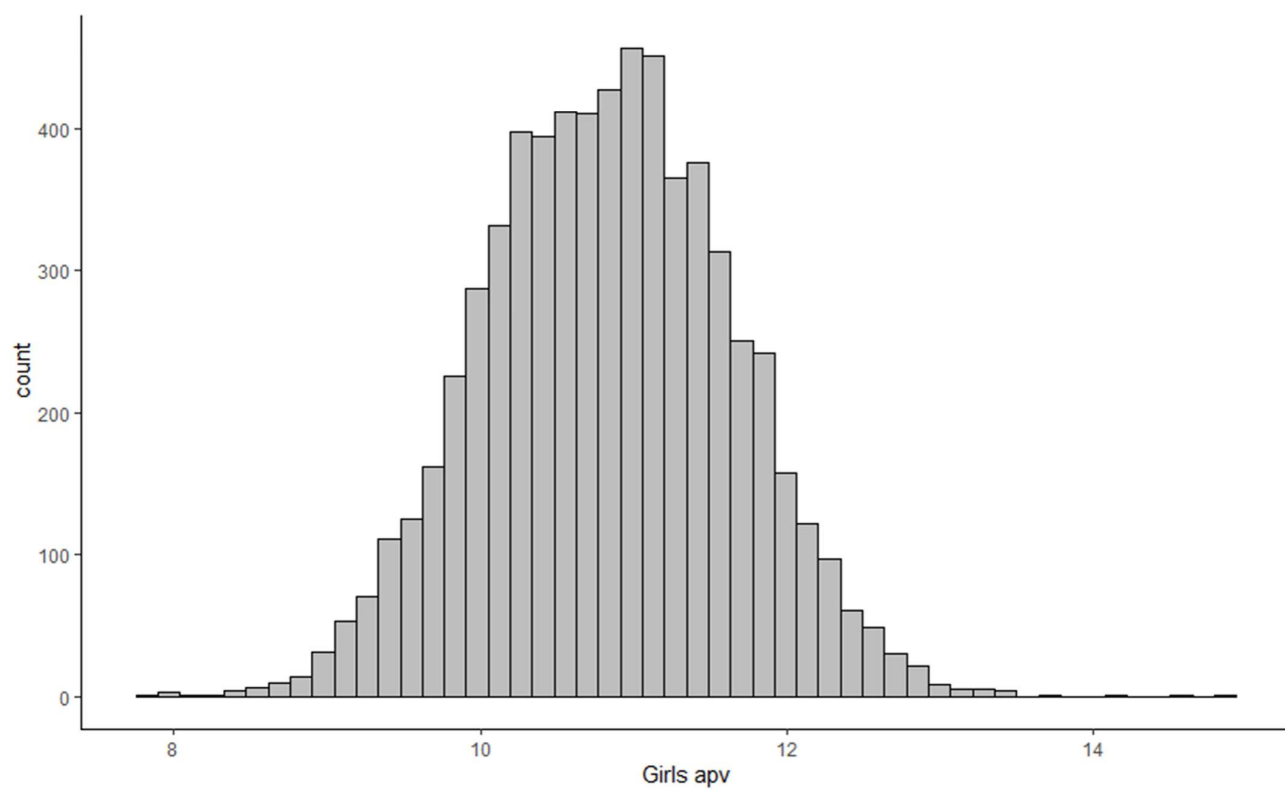

Supplement: Supplementary file 1 — Supplementary Figure 1 [file 41390_2023_2706_MOESM1_ESM.pdf]
