# Supplementary material for: Association between infant breastfeeding practices and timing of peak height velocity: A nationwide longitudinal survey in Japan
Source: Pediatr Res. 2023 Jul 3;94(5):1845–54. doi: 10.1038/s41390-023-02706-y (PMC10624627; doi:10.1038/s41390-023-02706-y)
Supplement: Supplementary file 2 — Supplementary Figure 2 [file 41390_2023_2706_MOESM2_ESM.pdf]

Supplemental Figure 2. The variance inflation factors for Table 3, Table 5, and Supplemental Table

VIF for Table 3

Variable	VIF	1/VIF
nut2		
2	4.49	0.222799
3	4.55	0.219593
BW		
1	1.52	0.656422
2	1.06	0.944184
wga		
1	1.39	0.717403
2	1.08	0.921823
1.multiple1	1.12	0.890584
bmi1	1.03	0.972040
mother_age~y		
1	2.85	0.351238
2	2.85	0.350656
mat_smoke_2		
1	1.02	0.984792
2	1.06	0.945242
99	1.00	0.998017
mat_edu_2		
1	1.93	0.517037
2	1.98	0.505497
3	1.18	0.846666
99	1.03	0.966543
city_3		
1	1.48	0.676044
2	1.49	0.670091
Mean VIF	1.80	

VIF for Table 5

Variable	VIF	1/VIF
nut_d		
2	3.67	0.272191
3	11.43	0.087477
4	12.64	0.079126
5	18.60	0.053759
6	14.46	0.069142
2.sex	1.01	0.994267
BW		
1	1.45	0.687583
2	1.08	0.924983
wga		
1	1.34	0.748205
2	1.11	0.901904
1.multiple1	1.12	0.895701
bmi1	1.03	0.967840
mother_age~y		
1	2.89	0.346340
2	2.89	0.345651
mat_smoke_2		
1	1.01	0.986280
2	1.08	0.925361
99	1.00	0.997809
mat_edu_2		
1	1.98	0.506200
2	2.03	0.491720
3	1.18	0.847768
99	1.05	0.955714
city_3		
1	1.51	0.663591
2	1.52	0.657338
Mean VIF	3.79	

VIF for supplemental Table 1

Variable	VIF	1/VIF
nut_d2		
3	3.63	0.275765
4	3.94	0.253811
5	5.43	0.184281
6	4.44	0.225007
2.sex	1.01	0.994269
BW		
1	1.45	0.687789
2	1.08	0.924986
wga		
1	1.34	0.748290
2	1.11	0.901979
1.multiple1	1.12	0.896342
bmi1	1.03	0.967909
mother_age~y		
1	2.89	0.346348
2	2.89	0.345798
mat_smoke_2		
1	1.01	0.986280
2	1.08	0.925423
99	1.00	0.997967
mat_edu_2		
1	1.98	0.506223
2	2.03	0.491800
3	1.18	0.847852
99	1.05	0.955759
city_3		
1	1.51	0.663596
2	1.52	0.657340
Mean VIF	1.99	
