# Supplementary material for: Association between infant breastfeeding practices and timing of peak height velocity: A nationwide longitudinal survey in Japan
Source: Pediatr Res. 2023 Jul 3;94(5):1845–54. doi: 10.1038/s41390-023-02706-y (PMC10624627; doi:10.1038/s41390-023-02706-y)
Supplement: Supplementary file 3 — Supplementary Table [file 41390_2023_2706_MOESM3_ESM.pdf]

Supplemental Table. Association between duration of breastfeeding and the APV (Formula-fed with/without colostrum as refer

APV		β*	Standard error	95% CI lower	95% CI upper	P-value
Formula-fed with/without colostrum(n=650)		(Reference)				
Breastfed 1–2 months (n=2413)		0.038	0.038	–0.036	0.11	0.31
3–5 months (n=2455)		0.076	0.037	0.003	0.15	0.04
6–7 months (n=4770)		0.1	0.036	0.033	0.17	0.004
Exclusively breastfed (n=2969)		0.12	0.037	0.051	0.2	0.001
Child characteristics						
Sex						
Male		(Reference)				
Female		–1.900	0.015	–1.930	–1.870	<0.001
Birth weight						
2500–4000 g		(Reference)				
<2500 g		–0.096	0.032	–0.160	–0.033	0.003
≥4000 g		0.086	0.08	–0.070	0.24	0.28
Weight						
Appropriate for gestational age		(Reference)				
Small for gestational age		–0.120	0.032	–0.180	–0.055	<0.001
Large for gestational age		0.072	0.024	0.024	0.12	0.003
Delivery						
Singleton birth		(Reference)				
Multiple birth		0.19	0.055	0.082	0.3	0.001
Prepubertal BMI		–0.120	0.005	–0.130	–0.110	<0.001
Maternal characteristics						
Age at delivery						
<25 years		(Reference)				
25–35 years		–0.050	0.03	–0.110	0.009	0.094
≥35 years		–0.089	0.034	–0.160	–0.021	0.01
Smoking habit						
None		(Reference)				
<10/day		–0.039	0.042	–0.120	0.043	0.35
≥10/day		–0.087	0.031	–0.150	–0.027	0.005
N/A		–0.330	0.13	–0.580	–0.082	0.009
Educational level						
University or higher		(Reference)				
Junior college or vocational school		–0.004	0.021	–0.044	0.037	0.86
High school		–0.020	0.022	–0.063	0.024	0.37
Junior high school or others		–0.120	0.051	–0.220	–0.023	0.016
N/A		0.057	0.08	–0.100	0.21	0.48
Residential area						
Ward		(Reference)				
City		–0.036	0.018	–0.072	0	0.05
Town or village		–0.047	0.024	–0.093	0	0.047

*Standardized regression coefficient

APV, age at peak height velocity; BMI, body mass index; CI, confidence interval; N/A, not applicable
